# Comprehensive analysis reveals TSPEAR as a prognostic biomarker in colorectal cancer

**DOI:** 10.7150/jca.90028

**Published:** 2024-01-01

**Authors:** Dong Xue, Hang Peng, Zhenghui Li, Jiarui Xu, Haiyun Ma, Yueyan Dang, Fanni Li, Guanghui Wang, Qi Sun

**Affiliations:** 1 Department of General Surgery, the First Affiliated Hospital of Xi'an Jiaotong University, Xi'an, Shaanxi, China.; 2 Department of Talent Highland, the First Affiliated Hospital of Xi'an Jiaotong University, Xi'an, Shaanxi, China.

**Keywords:** Colorectal cancer, TSPEAR, Prognosis, Immunity, Tumour mutation burden

## Abstract

**Background:** Colorectal cancer (CRC) is one of the most common malignant tumors and has high morbidity and mortality rates. Previous studies have shown that TSPEAR mutations are involved in the development and progression of gastric cancer and liver cancer. However, the role of TSPEAR in CRC is still unclear.

**Methods:** In The Cancer Genome Atlas (TCGA) database, 590 CRC patients with complete survival information were analyzed. We assessed TSPEAR expression in a pan-cancer dataset from the TCGA database. Cox regression analysis was performed to evaluate factors associated with prognosis. Enrichment analysis via the R package “clusterProfiler” was used to explore the potential function of TSPEAR. The single-sample GSEA (ssGSEA) method from the R package “GSVA” and the TIMER database were used to investigate the association between the immune infiltration level and TSPEAR expression in CRC. The R package “maftools” was used to explore the association between tumour mutation burden (TMB) and TSPEAR expression in CRC. CCK-8 assays and cell invasion assays were used to detect the effect of TSPEAR and TGIF2 on the biological behavior of CRC cells.

**Results:** Pan-cancer analysis revealed that TSPEAR was upregulated in CRC tissues compared to normal tissues and that high TSPEAR expression was associated with poorer overall survival (OS) (*p*=0.0053). The expression of TSPEAR increased with increasing TNM stage, T stage, N stage, and M stage. The nomogram constructed with TSPEAR, age, and TNM stage showed better predictive value than TSPEAR, age, or TNM stage alone. Immune cell infiltration analysis revealed that high expression of TSPEAR was associated with lower immune cell infiltration. Tumor mutation burden (TMB) analysis indicated that high expression of TSPEAR was associated with lower TMB (*p*=0.005), and high TMB was associated with shorter OS (*p*=0.02). CCK-8 assays and cell invasion assays indicated that in vitro knockdown of TSPEAR inhibited the proliferation, migration, and invasion of CRC cells. In addition, TSPEAR expression may be regulated by the upstream transcription factor TGIF2.

**Conclusion:** TSPEAR expression was higher in CRC tissues than in normal tissues. Its upregulation was significantly associated with a poor prognosis. Additionally, TSPEAR plays a significant role in tumor immunity and the biological behavior of CRC cells. Thus, TSPEAR may become a promising prognostic biomarker and therapeutic target for CRC patients.

## Introduction

According to global cancer statistics, the incidence rate of colorectal cancer (CRC) ranks third, and its mortality rate ranks second among cancers. It has been estimated that there were more than 1.9 million patients with CRC and 935,000 deaths in 2020, accounting for approximately one in ten cancer cases and deaths 1. At present, the diagnosis of CRC mainly depends on colonoscopy and biopsy. Common biomarkers of CRC, such as CEA and CA19-9, have low sensitivity and specificity, and cannot effectively diagnose CRC and monitor its recurrence. Despite rapid advances in diagnostic technology, most patients cannot be diagnosed early due to the lack of biomarkers for early detection, which leads to diagnosis in the advanced stage for some patients 2. There has been tremendous advancement in clinical treatments, including surgery, chemotherapy, radiotherapy, and immunotherapy. However, patients with CRC have limited benefits from these treatments 3. Thus, there is an urgent need to identify and apply new molecular targets for the diagnosis and treatment of patients with CRC 4.

The TSPEAR gene is located at 21q22.3, and its encoded protein is an extracellular protein. TSPEAR receptors are common on the cell surface. Previous studies have shown that TSPEAR mutation is related to the abnormal development of tooth and hair follicle morphology, which is mediated by the Notch signaling pathway 5. TSPEAR is broadly expressed in the liver, intestine, lung, kidney, and testis 6. Mutation and splicing of TSPEAR can participate in the development and progression of cancers, including gastric cancer and liver cancer 7. The downregulation of TSPEAR-AS1 expression in HBV-HCC may be a potential adverse prognostic factor. TSPEAR-AS1 may play an inhibitory role in HBV-HCC by inhibiting the proliferation, migration, and invasion of tumor cells 8. TSPEAR-AS1 plays a protective role in tongue squamous cell carcinoma. These findings indirectly indicate that SPEAR plays a carcinogenic role in some cancers 9. To date, there has been no report that TSPEAR is directly involved in the development and progression of cancer.

In our study, we analyzed the differential expression of TSPEAR between normal colorectal tissues and CRC tissues by utilizing RNA-seq data from The Cancer Genome Atlas (TCGA). Next, we investigated the relationship between the expression level of TSPEAR and clinical pathological features and analyzed the effect of TSPEAR on the prognosis of patients. Furthermore, we explored the potential mechanism of TSPEAR in the pathogenesis of CRC. Finally, experiments were performed to investigate the effects of TSPEAR and the transcription factor TGIF2 on the proliferation and invasion of CRC cells.

## Materials and Methods

### Dataset analysis

RNA-seq data of 590 CRC patients and their clinicopathological parameters were downloaded from the TCGA (https://portal.gdc.cancer.gov/) database. CRC associated with clinical information includes age, gender, tumor location, clinical tumor stage, T stage, N stage, M stage, and live status. Patients with incomplete clinical information were excluded. In addition, we downloaded information on normal tissue samples from the Genotype-Tissue Expression (GTEx) (https://www.gtexportal.org/) database.

### Human tissue samples

We collected 10 samples of CRC in the First Affiliated Hospital of Xi'an Jiaotong University, China. The Ethics Committee for Clinical Trials of the First Affiliated Hospital of Xi'an Jiaotong University approved this research. All patients signed informed consent. The detailed including criteria for the enrolled patients in this study are: (1) all patients have been diagnosed with CRC adenocarcinoma according to WHO criteria; (2) patients did not have other tumors or diseases; (3) patients did not receive radiation treatment and chemotherapy drugs. Meanwhile, the adjacent CRC tissues were obtained from patients during surgery. All the samples were immediately frozen in liquid nitrogen and prepared for RNA extraction.

### Survival prognosis analysis

In this study, univariate and multivariate Cox regression analyses and forest maps were used to study the influence of the TSPEAR gene and clinicopathological characteristics on CRC prognosis, and the *p* value, HR, and 95% CI of each variable were displayed via the R package “forestplot”. "Survival" and "survminer" packages are commonly used in analyzing survival. The "survival" package is responsible for the analysis, and the "survminer" package visualizes the analysis results. Selecting the median value of TSPEAR as the dividing threshold, these patients were divided into the high-expression group and low-expression group. We used the R packages “survival” and “survminer” to generate the overall survival (OS) and disease-specific survival (DSS) curves. Additionally, we evaluated the capacity of TSPEAR to distinguish CRC tissues from normal tissues via receiver operating characteristic (ROC) curve analysis.

### Construction and validation of a nomogram

Patients were divided into a training set and a validation set at a ratio of 7:3. The "rms" package is the most commonly used drawing tool for nomograms and calibration plots, and it is widely used in nomograms. We chose the variables with *p* < 0.05 in the multivariate Cox analysis to construct a nomogram. Based on the nomogram, we calculated the total risk score. The median value of the total risk score was used as the cut-off point, according to which CRC patients were divided into a high-risk group and a low-risk group; we subsequently performed Kaplan‒Meier survival analysis. We used the R package “survivalROC” to draw ROC curves to compare the predictive power of the nomogram and other clinical variables. The calibration plots were drawn using the R package “rms”.

### Immune cell infiltration analysis

CIBERSORT is the most frequently used immune cell infiltration analysis tool, which provides comprehensive immune cell types, including 22 immune cells, and provides accurate immune cell infiltration analysis. CIBERSORT software was used to calculate the proportion of 22 types of immune cells in each sample to investigate the correlation between TSPEAR and the abundance of tumor-infiltrating immune cells. The *p* value was calculated using the Wilcoxon rank-sum and Spearman's rank correlation tests. Gene Set Variation Analysis (GSVA) is mainly used to evaluate the gene set enrichment results of chips and transcriptomes. By transforming the expression matrix of genes between different samples into the expression matrix of gene sets between samples, it is possible to evaluate whether different metabolic pathways are enriched between different samples. ssGSEA can quantify the abundance of immune cell infiltration in each sample, and it is a commonly used analysis method of immune cell infiltration. We performed ssGSEA to investigate the difference in immune infiltrating cells and immune function enrichment between the high-expression group and the low-expression group. In addition, the differences in stromal scores, immune scores, and ESTIMATE scores were compared between the high-expression group and the low-expression group and displayed in boxplots. We performed an immunotherapy response-related analysis using the IMvigor210 cohort, which included 348 samples that were immunotherapy treated and included immunotherapy response status and survival information.

### Correlation analysis between TSPEAR and TMB

The heterogeneity and complexity of cancer has always been major limitation for the treatment and research of cancer. The study of tumor mutation spectrum can show the high molecular heterogeneity among cancers. Maftool is a powerful tool for analyzing tumor mutation data. The top 20 most commonly mutated genes of the low and high expression groups were compared with the R package “maftools” and visualized via waterfall plots. The TMB of the two groups was compared and displayed via boxplots. Based on survival analysis, we investigated the influence of TMB on the OS of CRC patients.

### Analysis of differential expression

The “limma” package is an algorithm based on voom, which can perform difference analysis on both chip data and high-throughput sequencing data, and has obvious advantages in merging TCGA and GTExs data. Differentially expressed genes (DEGs) between the high expression group and low expression group were identified using the R package “limma”. According to the following criteria: false discovery rate (FDR) < 0.05 and |log_2_-fold change (FC)| > 1, we used the R package “ggplot2” to draw the volcano plot of DEGs. ClusterProfiler is a universal enrichment analysis tool, that supports GO, KEGG, and GSEA enrichment analysis, and can easily visualize the enrichment analysis results. The R package “clusterProfiler” was used to perform Gene Ontology (GO) and Kyoto Encyclopaedia of Genes and Genomes (KEGG) analyses.

### Screening of the transcription factor associated with TSPEAR

According to the thresholds of read count median > 1 and average > 10, highly expressed transcription factors were identified in the TCGA-COADREAD dataset. Based on the thresholds of |log_2_ FC| > 1 and q < 0.05, differentially expressed transcription factors were identified between CRC tissues and normal tissues. We investigated the correlation between transcription factors and TSPEAR using Spearman correlation analysis. The 3000 bp sequence file for the region upstream of the start site of the TSPEAR gene was downloaded from the UCSC database. The motif files corresponding to the transcription factors were obtained from the JASPER database. FIMO online analysis tool is used to determine all the matching positions of transcription factor motif in one or more promoter sequences, and accurately predict the binding position of each transcription factor in each Chip-seq peak. The FIMO was used to predict whether there is a transcription factor binding motif in the upstream region of the TSPEAR promoter. The ChIP-seq public database Cistrome was used to predict the transcription factor binding peak.

### Cell culture

The human CRC cells (HT29, HCT116, SW480, and SW620) and normal colonic mucosa cell (NCM460) were procured from the Shanghai Cell Bank of Chinese Academy of Sciences (Shanghai, China). Cells were cultured on DMEM or RPMI 1640 medium (Gibco, USA) supplemented with 10% fetal bovine serum (FBS) (Gibco, USA). The medium was placed in a 37°C, 5% CO2 incubator for stationary culture to reach 80-90% density.

### RNA interference and transfection

The small interfering RNAs of TSPEAR were obtained from Shanghai GenePharma Co. Ltd (Shanghai, China). And 50 nmol/L siRNA was transfected into HCT116 and HT29 cells by using Lipofectamine 2000 (ThermoFisher, Massachusetts, USA). Knockdown efficiency was evaluated by qPCR. The sequences of siRNA were: TSPEAR, SS- 5′- CCUUCUCGGUGAACAGUAUTT -3′, AS- 5′- ATACTGTTCACCGAGAAGGTT -3′. TGIF2, SS- 5′-GGAUGGCAAAGACCCUAAUTT -3′, AS- 5′- AUUAGGGUCUUUGCCAUCCTT -3′.

### Real-time quantitative PCR

Total RNA was extracted from cells or tissues by using an RNAfast200 kit (Fastagen, Shanghai, China). cDNA library was constructed by using PrimeScriptTM RT Master Mix (Takara, Shiga, Japan). qPCR was performed on an ARIAMX system (Agilent, USA) with a 10 μl reaction mixture containing SYBR GreenII. GAPDH was used as an endogenous reference gene and the result was utilized by the 2^^-△△CT^ method. The primer sequences were: GAPDH, F-5′-TCAGCAATGCCTCCTGCAC-3′, R-5′-TCTGGGTGGCAGTGATGGC-3′. TSPEAR, F-5′-CGGTGGACATAATGGCCGAT-3′, R-5′-AGCACCTCGTTATCTTCTGGC-3′.

### Western blot analysis

Total protein was extracted from colon cancer cells with RIPA lysis buffer containing protease inhibitor (Beyotime, Beijing, China). Protein concentration was quantified with a BCA kit (Beyotime, Beijing, China). The protein was separated with 10% SDS-PAGE and transferred to PVDF membranes. After blocked for 1 h with skimmed milk, the membrane was incubated with the primary anti-TSPEAR antibody (1:1000) (Proteinch, Wuhan, China) at 4℃ overnight. Then the membrane was incubated with HRP-conjugated secondary antibody (1:10000) (Proteintech, Wuhan, China) at room temperature for 2 h. GAPDH (1:1000) (Proteintech, Wuhan, China) is endogenous for normalization and ECL was performed to obtain visualized images.

### Cell counting kit-8 (CCK-8) analysis

CCK-8 assay was performed to detect the reproductive ability of cells. Specifically, HCT116 and HT29 cells were cultured in a 96-well plate at a density of 2000 cells per well. At 24 h, 48 h, 72 h, and 96 h, 10 μl CCK-8 solution (Beyotime, Beijing, China) was added to each well, and the cells were incubated at 37℃ for 90 min. The absorbance at 450 nm was detected using an enzyme-labeled instrument. Graphpad Prism 8 version software was used to display the cell proliferation curve.

### Cell invasion assay

After thawing, the matrigel (Corning, New York, USA) was uniformly mixed with the pre-cooled pipette head. The culture plates were placed on ice in advance, and matrigel with a concentration of 50 μl/cm^2^ was added and placed at 37℃ for 30 min. The tumor cells were prepared into cell suspension with a concentration of 10^5^ cells/ml, and 300 μl cell suspension was added into the upper chamber. 1.5 ml DMEM with 10% FBS was added to the lower chamber. The cells were cultured at 37℃ in a 5% CO_2_. After 48 h, the upper chamber was fixed with paraformaldehyde for 20 min and stained with crystal violet for 30 min. Non-invading cells on the upper chamber were removed with a cotton swab, and the cells on the lower surface were counted and photographed under a microscope.

### Statistical analysis

The Wilcoxon rank sum test was used to detect the difference in gene expression and immune cell infiltration between subgroups. Cox regression and Kaplan-Meier analysis were employed in the survival study. Log-rank test was used to test the significance of the survival rate difference. Kruskal-Wallis tests were used to evaluate the relationship between TSPEAR expression and clinical stage. ROC was used to assess the discriminative power of TSPEAR. Spearman's correlation coefficient was used in correlation analysis. *T* tests were used for paired samples. *P* values < 0.05 were considered to indicate statistical significance. All of the above statistical analyses were performed using R software version 4.2.1.

## Results

### TSPEAR is upregulated in the cancer tissue of CRC patients

**Figure 1:**
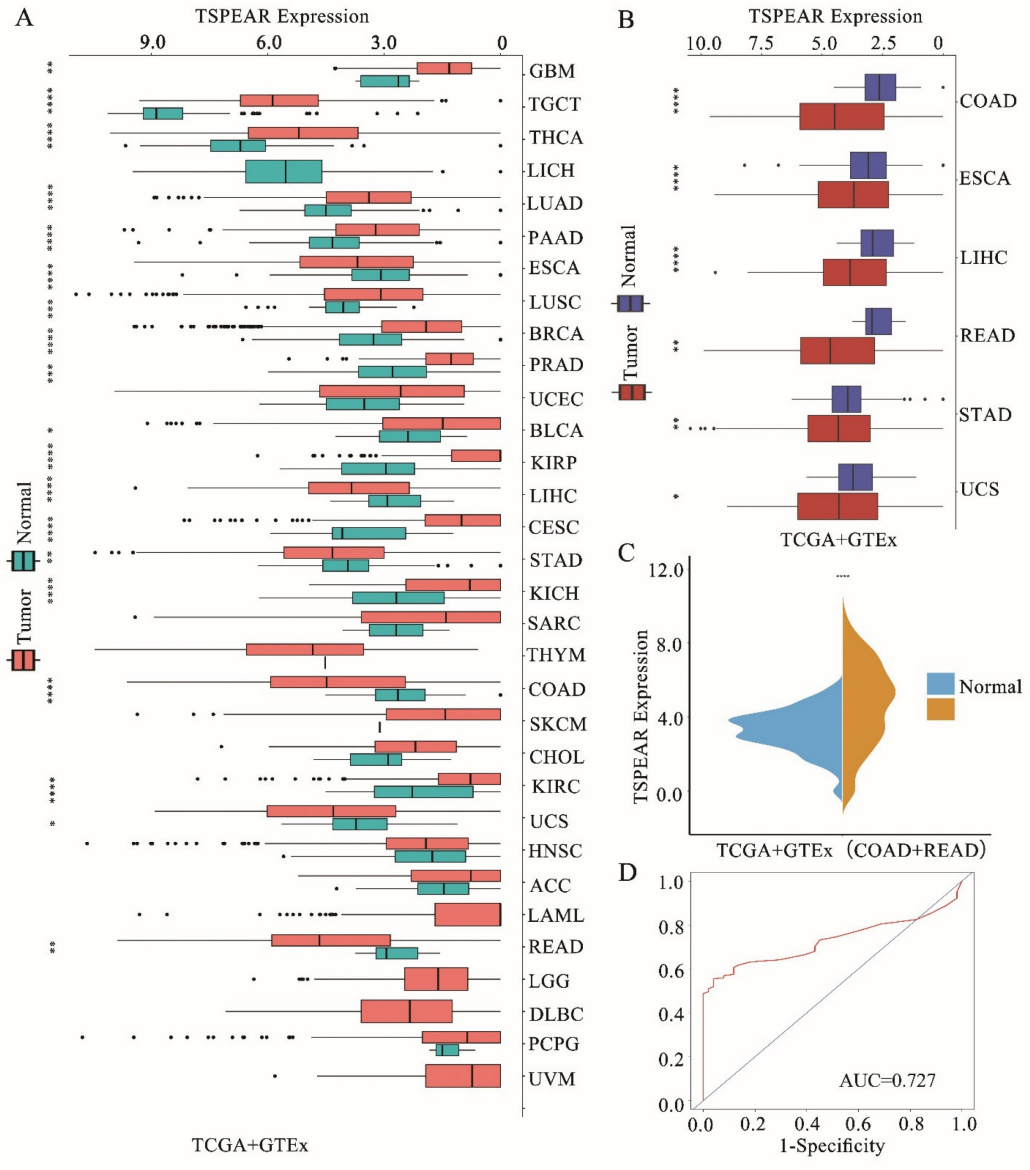
** Expression of TSPEAR in CRC. (A)** Expression of TSPEAR in 32 types of cancers. *P* values are shown as *p<0.05; **p<0.01; ***p<0.001. **(B)** TSPEAR is overexpressed in 6 types of cancers. **(C)** TSPEAR is overexpressed in CRC. **(D)** ROC curve of TSPEAR in CRC. The X-axis represents false-positive rates, and the Y-axis represents true-positive rates.

To explore the expression of TSPEAR in various tumor tissues, we combined the TCGA database with the GTEx database to explore the expression of TSPEAR in cancer tissues and normal tissues. The results showed that TSPEAR was highly expressed in some cancers, including ESCA, LIHC, STAD, COAD, UCS, and READ. However, TSPEAR was expressed at low levels in most cancers (Figure 1A, B). By searching the reported studies, we found that TSPEAR was rarely studied in CRC. Therefore, we further analyzed the expression of TSPEAR in CRC based on TCGA and GTEx databases (Figure 1C). Additionally, we evaluated the ability of TSPEAR to distinguish CRC tissues from normal tissues by ROC curve analysis. The area under the ROC curve (AUC) was 0.727, displaying a favorable ability of discernment (Figure 1D).

### Evaluation of the prognostic relevance of TSPEAR in CRC

**Figure 2:**
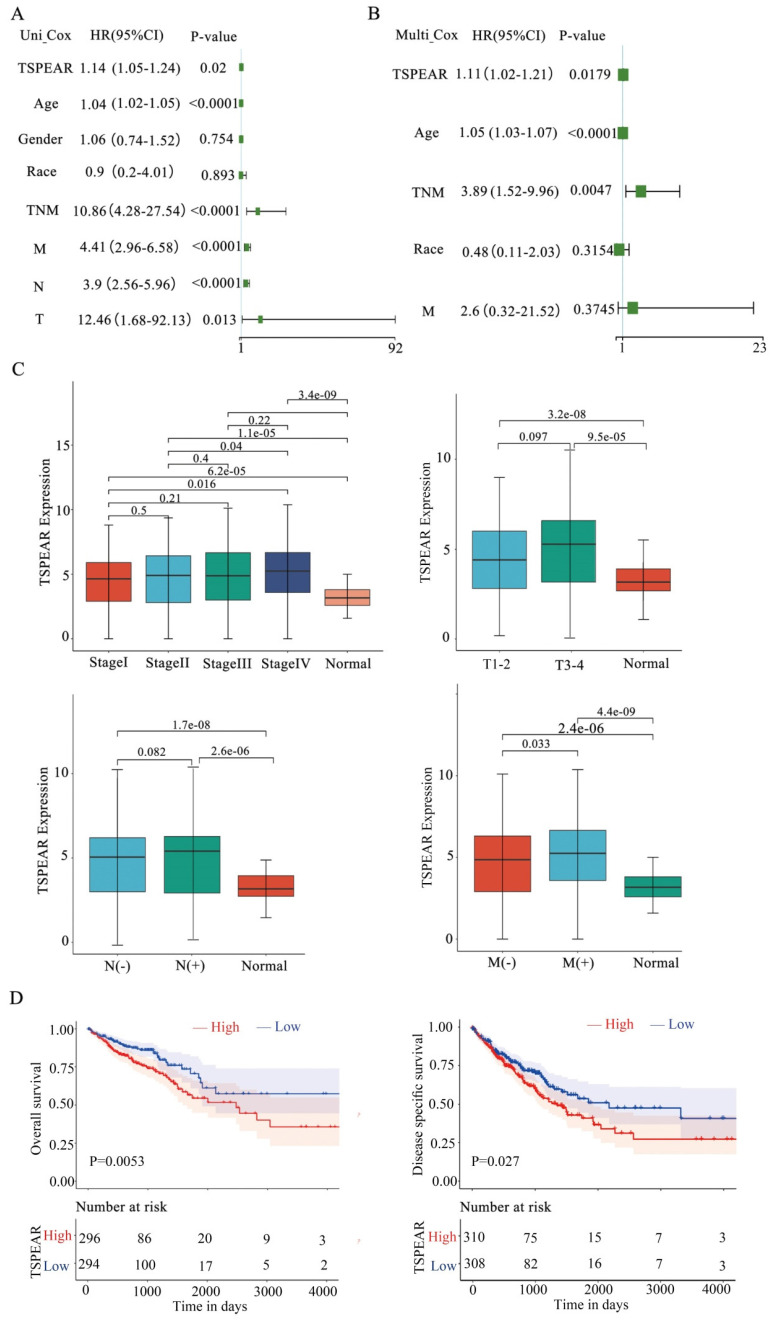
** High expression of TSPEAR indicates poor survival in patients with CRC. (A)** The forest plots show that the risk factors for the overall survival of CRC were analyzed by univariate Cox regression analysis. **(B)** The forest plots show that the risk factors for overall survival of CRC were analyzed by multivariate Cox regression analysis. **(C)** Correlation between TSPEAR expression and tumor stage in CRC. **(D)** Kaplan‒Meier curves were used to analyze the influence of TSPEAR on OS and DSS in CRC patients.

We explored the significance of TSPEAR in the progression of CRC. 590 CRC patients and their clinicopathological parameters were shown (Table 1). Univariate and multivariate Cox regression analyses were used to investigate the relationship between TSPEAR, clinical factors (age, sex, race, TNM stage, T stage, N stage, and M stage), and OS in CRC patients. Univariate Cox regression analysis showed that TSPEAR (*p* = 0.02), age (*p* < 0.0001), TNM stage (*p* < 0.0001), T stage (*p* = 0.013), N stage (*p* < 0.0001) and M stage (*p* < 0.0001) were significantly associated with OS in CRC patients (Figure 2A). Multivariate Cox regression analysis indicated that TSPEAR (*p* = 0.0179), age (*p* < 0.0001), and TNM stage (*p* = 0.0047) were independent prognostic factors for CRC patients (Figure 2B).

We found that the expression of TSPEAR increased with the TNM stage, T stage, N stage, and M stage (Figure 2C). The median value of TSPEAR expression was the cut-off point, according to which CRC patients were divided into high and low-expression groups (Figure S1). Kaplan‒Meier (KM) survival analysis indicated that the high TSPEAR group had shorter OS (*p* = 0.0053) and DSS (*p* = 0.027) (Figure 2D). Thus, TSPEAR plays an important role in the progression of CRC. The ROC curves of 1-, 2- and 5-year survival showed that TSPEAR *was insufficiently accurate* as a predictive marker (AUC = 0.59) (Figure S2).

### Construction of a nomogram

**Figure 3:**
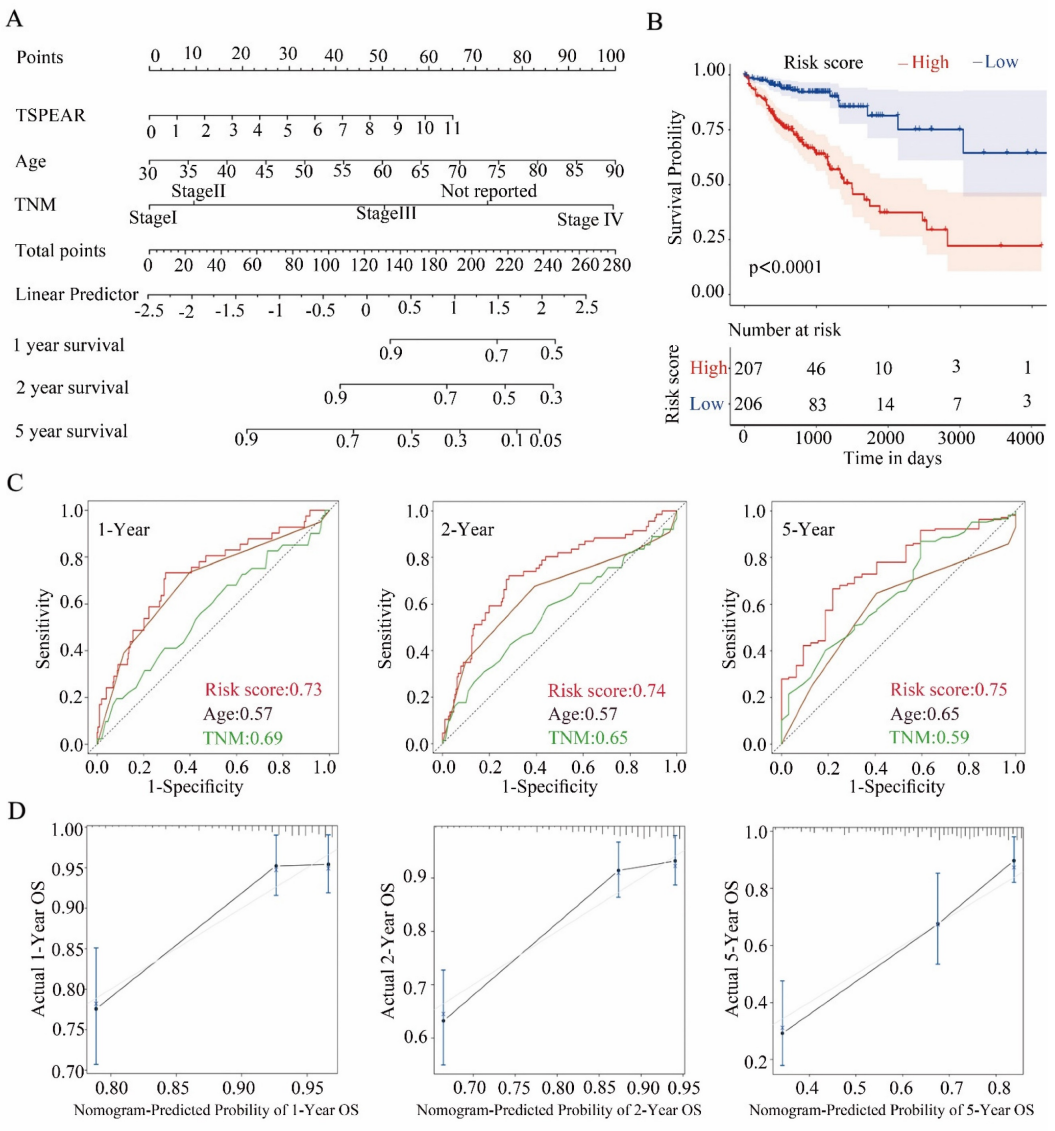
** Construction of a nomogram in the training cohort. (A)** The nomogram consists of TSPEAR, age, and TNM stage. **(B)** Kaplan‒Meier curves were used to analyze the relationship between risk score and OS based on the nomogram. **(C)** ROC curves of 1-, 2-, and 5-year OS predicted by the nomogram. **(D)** Calibration curves of 1-, 2-, and 5-year OS predicted by the nomogram.

To better predict the OS of CRC patients, we chose the significant variables with *p* < 0.05 in the multivariable Cox analysis, including TSPEAR, age, and TNM stage, to construct a nomogram (Figure 3A). According to the nomogram, the total risk score was calculated. Selecting the median value of the total risk score as the cut-off point, patients were classified into a high-risk group and a low-risk group. KM survival analysis showed that OS was better in the low-risk group than in the high-risk group (Figure 3B). The ROC curves for 1-, 2- and 5-year survival also showed that the nomogram predicted OS better than age and TNM stage (Figure 3C). We used the calibration curve to evaluate the predictive value of the nomogram, and the results indicated strong consistency between the nomogram prediction and the actual observation (Figure 3D).

### Validation of the nomogram in the internal validation cohort

**Figure 4:**
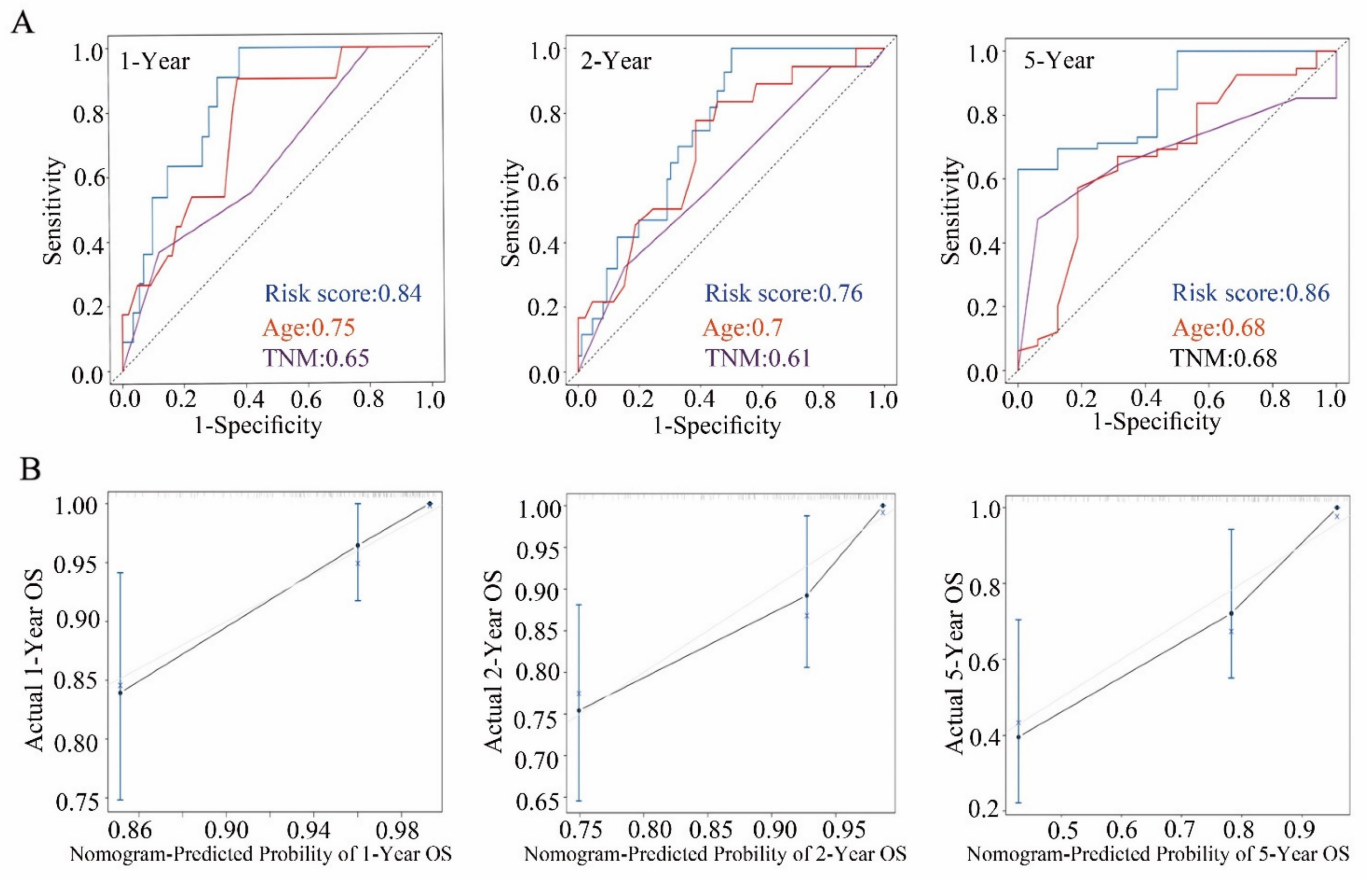
** Construction of a nomogram in the internal validation cohort. (A)** ROC curve of 1-, 2-, and 5-year OS predicted by the nomogram.** (B)** Calibration curves of 1-, 2-, and 5-year OS predicted by the nomogram.

We used the TCGA internal validation cohort to test the validity of the nomogram via the same process. Based on the same formula, we classified patients into a high-risk group and a low-risk group. As expected, the high-risk group had significantly shorter OS than the low-risk group (Figure S3). The ROC curves of 1-, 2- and 5-year survival also showed that the nomogram had better predictive ability (Figure 4A). The calibration curves for 1-, 2- and 5-year survival indicated that the nomogram predictions were strongly consistent with the actual observations (Figure 4B).

### Low immune cell infiltration in the high-expression group

**Figure 5:**
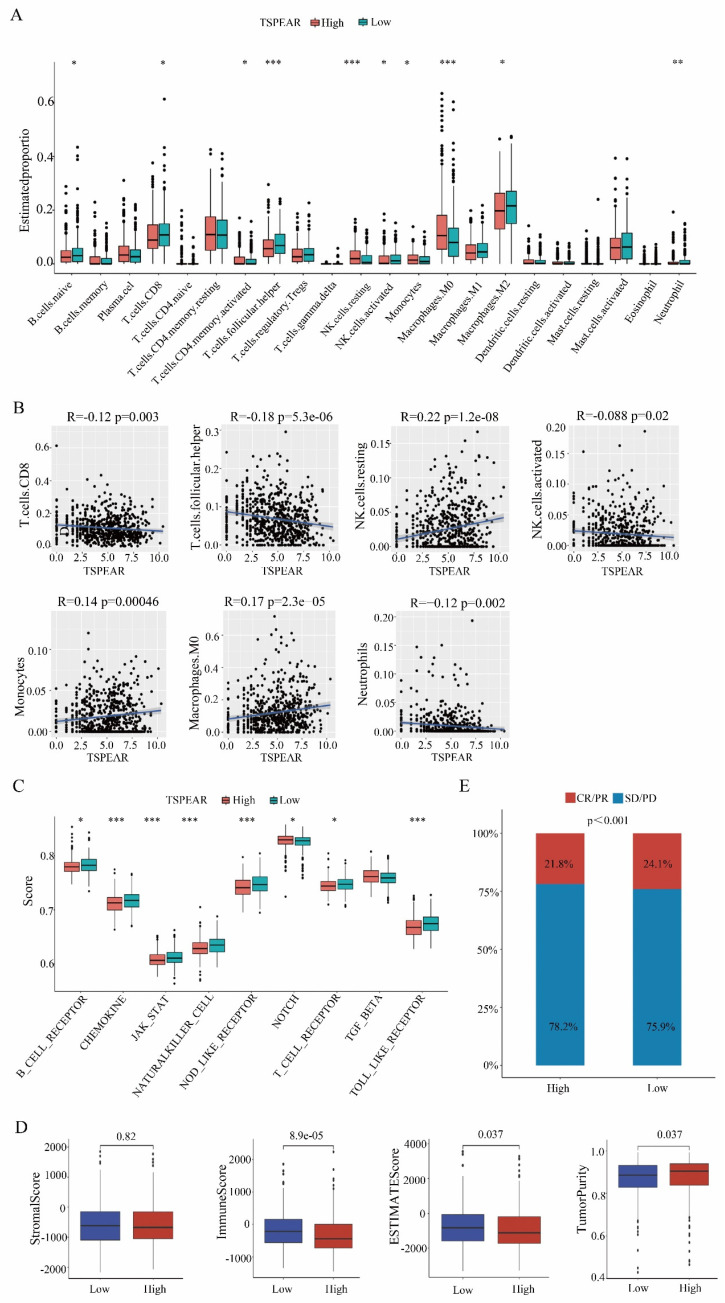
** Correlation between TSPEAR and immune infiltration. (A)** Comparison of the infiltration proportion of 22 types of immune cells between the high-expression group and the low-expression group.** (B)** Correlation between TSPEAR expression and the levels of CD8 T cells, T follicular helper cells, resting NK cells, activated NK cells, monocytes, M2 macrophages, and neutrophils. **(C)** Comparison of the enrichment of 9 immune-related pathways between the high expression group and the low expression group.** (D)** Stromal score, immune score, ESTIMATE score, and tumor purity in the high and low expression groups. **(E)** Comparison of the immunotherapeutic effect in the high and low expression groups.

Immune cells are the main cellular components in local tumor lesions, but there are great differences in the types and functions of infiltrated immune cells in different tumor microenvironments. In our study, we used CIBERSORT software to display the proportions of 22 types of immune cells in each sample (Figure S4). Furthermore, we examined the relationship between TSPEAR and immune infiltration by ssGSEA, and the results showed significant differences in the levels of infiltrating immune cells, including naïve B cells and T cells. CD8 T cells, activated memory CD4 cells, follicular helper T cells, resting NK cells, activated NK cells, monocytes, M0 macrophages, M2 macrophages, and neutrophils. The immune cell infiltration levels of naïve B cells, CD8 T cells, memory-activated CD4 T cells, follicular Helper T cells, activated NK cells, M2 macrophages, and neutrophils were lower in the high expression group than in the low expression group (Figure 5A). We also assessed the potential correlation between TSPEAR and immune cell infiltration using Spearman's rank correlation analysis. The results showed that TSPEAR expression was positively correlated with the levels of resting NK cells (R = 0.22, *p* = 1.2e-08), monocytes (R = 0.14, *p* = 0.00046), and M0 macrophages (R = 0.17, *p* = 2.3e-05). In contrast, TSPEAR expression was negatively associated with the levels of CD8 T cells (R = -0.12, *p* = 0.0034), follicular helper T cells (R = -0.18, *p* = 5.3e-0.6), activated NK cells (R = -0.088, *p* = 0.026) and neutrophils (R = -0.12, *p* = 0.0027) (Figure 5B).

### Low enrichment scores of immune-related pathways in the high-expression group

We investigated the enrichment scores of immune-related pathways between the high-expression and low-expression groups. Compared to the low expression group, the high expression group had lower enrichment scores of immune-related pathways, including B-cell receptor, chemokine, JAK-STAT, natural killer cell, NOD-like receptor, NOTCH, T-cell receptor, and TOLL-like receptor pathways (Figure 5C). In the tumor microenvironment, immune and stromal cells are two major types of non-tumor components and have been proposed to be valuable for the diagnosis and prognosis evaluation of tumors. In our study, we used the ESTIMATE method to calculate the stromal score, immune score, ESTIMATE score, and tumor purity for the high-expression and low-expression groups. The immune score and ESTIMATE score were lower in the high expression group than in the low expression group. Tumor purity was higher in the high-expression group than in the low-expression group. There was no difference in stromal scores between the high-expression and low-expression groups (Figure 5D). Correlation analysis of the immunotherapeutic response in the IMvigor210 cohort revealed that high TSPEAR expression was associated with a low level of patient immune response (Figure 5E). These results suggested that the immune infiltration level was lower in the high-expression group than in the low-expression group.

### High TMB in the high-expression group

**Figure 6:**
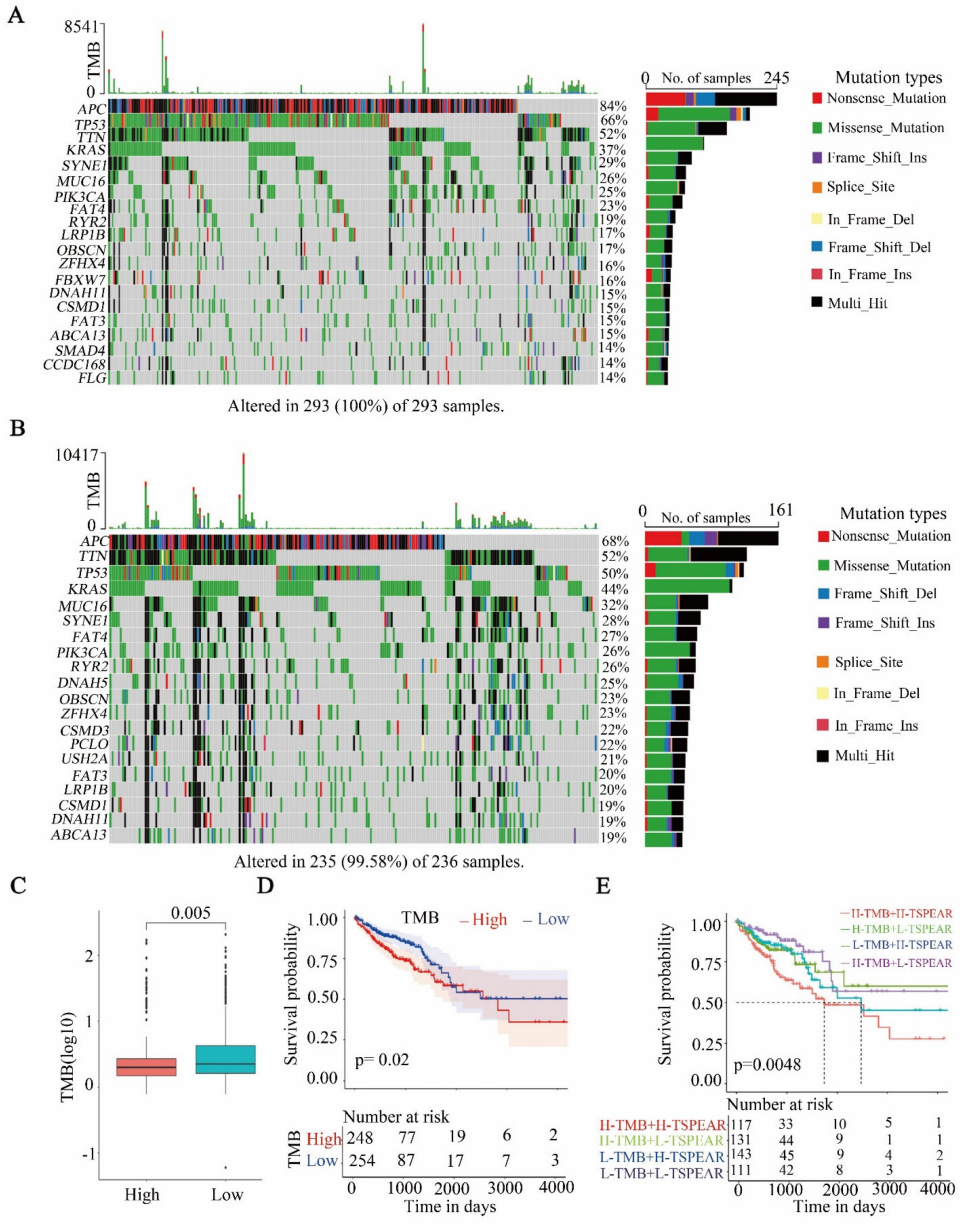
** Correlation between TSPEAR and TMB. (A)** Waterfall plot of the top 20 mutated genes in the high-expression group.** (B)** Waterfall plot of mutations in the low expression group.** (C)** Comparison of TMB between the high expression group and the low expression group.** (D)** Survival analysis between the high and low TMB cohorts.** (E)** Survival analysis for patients considering TMB and TSPEAR.

After dividing the samples into two groups based on the TSPEAR expression level and evaluating somatic mutation information, the differences in the top 20 mutated genes between the high-expression and low-expression groups were shown via waterfall plots (Figure 6A, B). The results showed that the most commonly mutated gene was APC (high: 84%, low: 68%). Compared to the low-expression group, the high-expression group exhibited a significantly lower TMB (Figure 6C). Survival analysis showed that patients with higher TMB had shorter OS than those with lower TMB (Figure 6D). Furthermore, we combined TSPEAR expression with TMB for survival analysis, and the results showed that high levels of TSPEAR and TMB were associated with a poor prognosis in CRC patients (Figure 6E).

### Correlation and TSPEAR-related gene enrichment analysis

**Figure 7:**
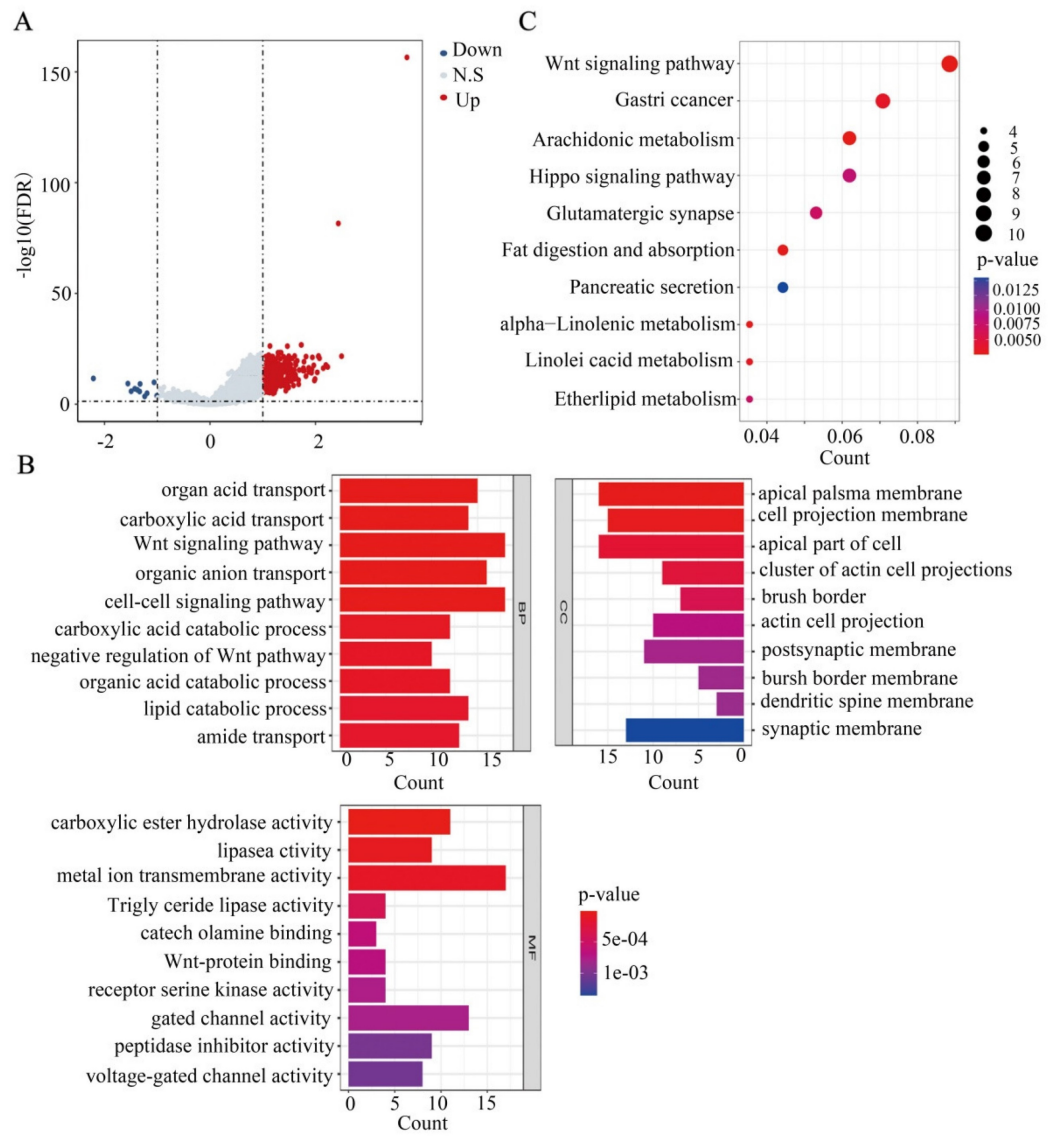
** Functional analysis based on the DEGs between the high expression group and the low expression group. (A)** DEGs are shown in a volcano plot (blue: downregulated genes; grey: none; red: upregulated genes).** (B)** GO enrichment analysis of DEGs.** (C)** KEGG pathway analysis of DEGs.

In this study, we explored the potential function of DEGs between the high-expression and low-expression groups. Based on the criteria of FDR < 0.05 and |log_2_ (FC)| > 1, 344 genes were identified between the high-expression and low-expression groups, including 323 upregulated genes and 21 downregulated genes (Figure 7A). Furthermore, GO term annotation showed that DEGs were mainly enriched in organic acid transport, the Wnt signaling pathway, cell-cell signalling, and other biological processes (Figure 7B). KEGG pathway analysis indicated that DEGs were mainly enriched in the Wnt signaling pathway, Hippo signaling pathway, gastric cancer, and other pathways (Figure 7C). These results suggested that TSPEAR may regulate the biological behavior of CRC through multiple signaling pathways.

### Transcription factor TGIF2 regulates the expression of TSPEAR

**Figure 8:**
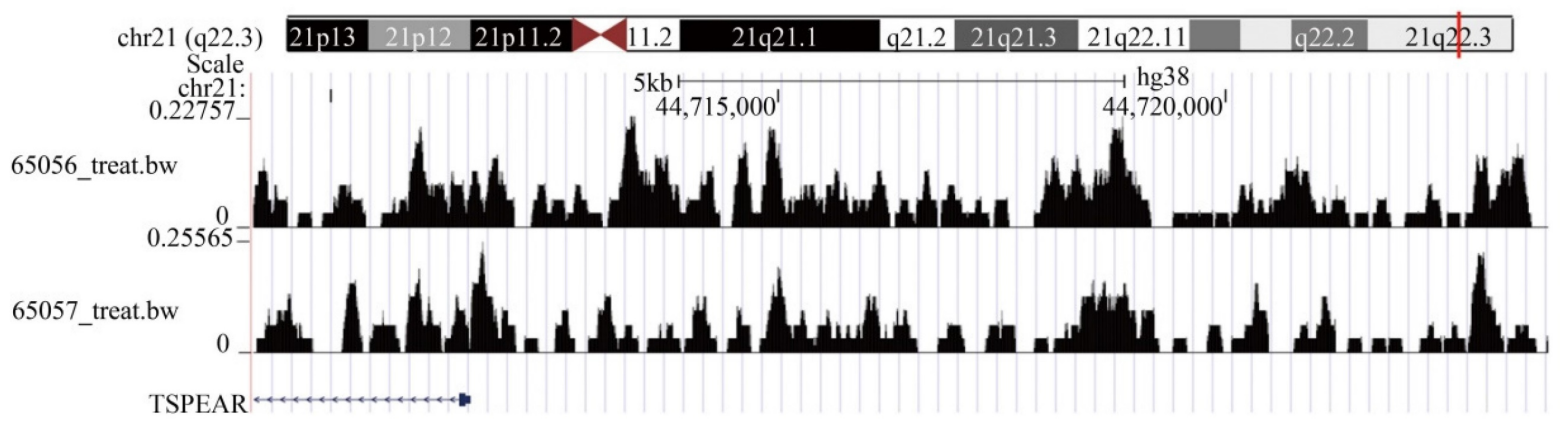
TGIF2 ChIP-seq showed a significant binding peak on TSPEAR.

In addition, we analyzed the upstream transcription factors that regulate TSPEAR expression. A total of 263 differentially expressed and highly expressed transcription factors were identified between CRC tissues and normal tissues. Based on the criteria of *p* < 0.05 and Rho > 0.3, we found that 51 transcription factors were positively associated with TSPEAR expression (Table S1). The motif of positively correlated transcription factors was compared with the sequence in the promoter region of the TSPEAR gene (3000 bp upstream of the TSPEAR gene). With *p* < 10^-4^ as the threshold, we found that the PLAGL2 binding site may be present approximately 50 bp upstream of TSPEAR, and the TGIF2 binding site may be present approximately 241 bp upstream of TSPEAR (Table S2). Through the ChIP-seq public database Cistrome, we found that TSPEAR showed a significant TGIF2 binding peak in ENCSR993LMB_-_1 and ENCSR993LMB_-_2. These results indicated that the transcription factor TGIF2 could regulate TSPEAR expression by binding the upstream sequence of the TSPEAR promoter (Figure 8).

### TSPEAR knockdown inhibited cell proliferation, migration, and invasion

**Figure 9:**
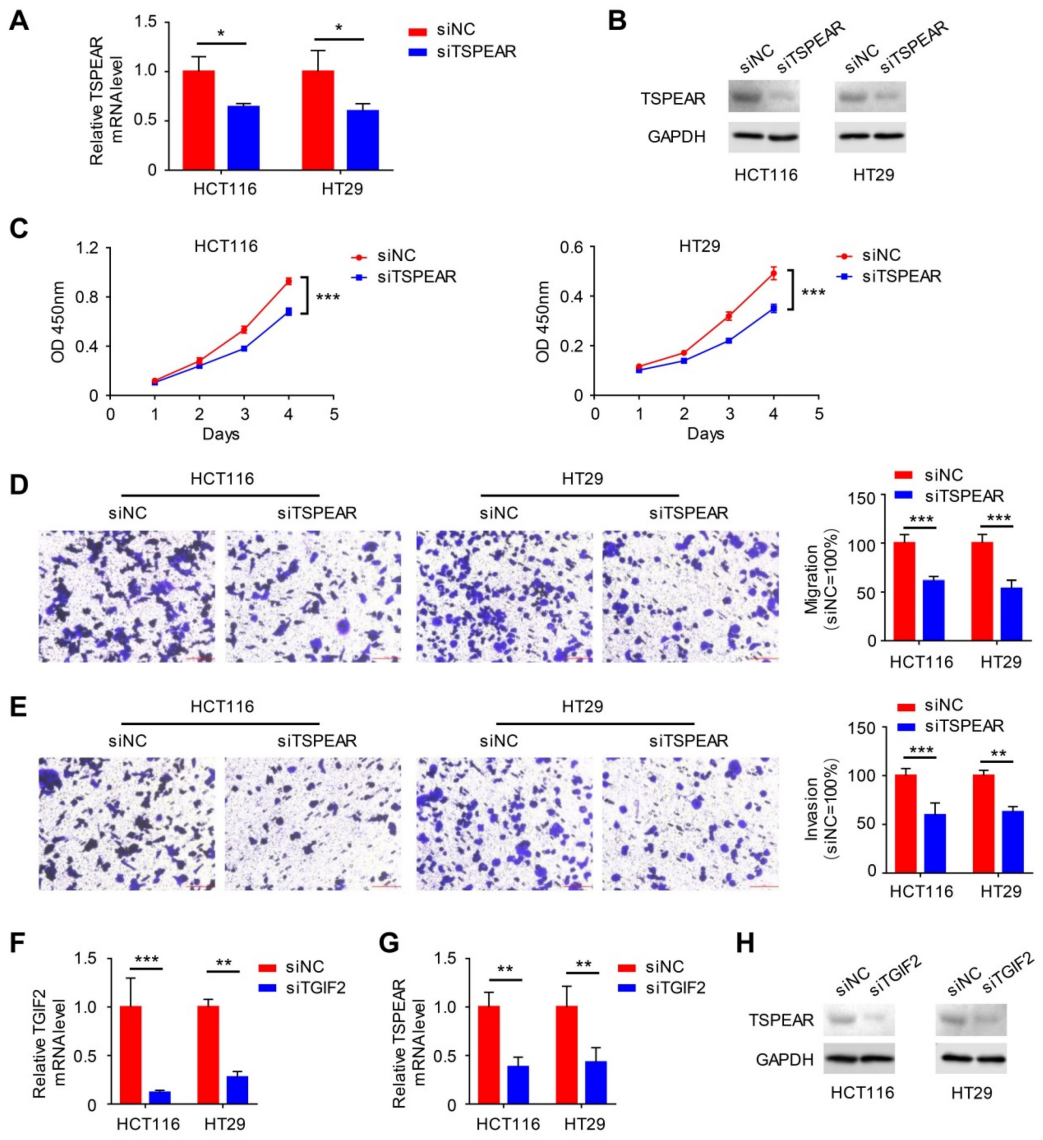
** Effect of TSPEAR and TGIF2 on the biological behavior of colon cancer cells. (A)** siRNA interference of the expression of TSPEAR in HT29 and HCT116 cells.** (B)** Western blotting was performed to detect the TSPEAR protein level after siTSPEAR interference.** (C-E)** Effects of TSPEAR on the proliferation ability of HT29 and HCT116 cells. *P* values are shown as ***p<0.001; effect of TSPEAR on the migration ability of HT29 and HCT116 cells. The *P* value is shown as ***p<0.001.** (F)** The relative TGIF2 mRNA level after siTGIF2 interference. *P* values are shown as **p<0.01; ***p<0.001.** (G, H)** The relative TSPEAR mRNA level after siTGIF2 interference. The *P* value is shown as **p<0.01; Western blotting was performed to detect TSPEAR after siTGIF2-mediated interference.

We detected the mRNA expression levels of TSPEAR in CRC specimens and their paired normal tissues. The results showed that the mRNA expression level of TSPEAR was much higher in cancer tissues than that in normal tissues (Figure S5). Additionally, we examined the mRNA expression levels of TSPEAR in the normal colonic mucosa cell line NCM460 and CRC cell lines HT29, SW480, SW620, and HCT116. The results indicated that TSPEAR was expressed at higher levels in CRC cells compared to normal colonic mucosa cells (Figure S6). To further explore the effect of TSPEAR on the biological function of CRC cells, HT29 and HCT116 cell lines with the highest expression of TSPEAR were used for in vitro experiments. We used siTSPEAR to inhibit TSPEAR expression in HCT116 and HT29 cells, and the results showed that the relative TSPEAR mRNA level was lower in the siTSPEAR group than in the siNC group (Figure 9A). Western blot results showed that TSPEAR protein levels were lower in the siTSPEAR group than in the siNC group (Figure 9B). Compared to the siNC group, cell proliferation, migration, and invasion were inhibited in the siTSPEAR group (Figure 9C-E). Similarly, we knocked down TGIF2 using siTGIF2 (Figure 9F). After knocking down TGIF2, we found that the relative TSPEAR mRNA and protein expression levels were decreased (Figure 9G, H).

## Discussion

CRC remains a global medical problem with high morbidity and mortality rates. The main causes of high mortality in patients with CRC are the lack of early diagnosis methods and the high degree of malignancy of CRC. Early diagnosis and effective treatment can significantly improve survival in patients with CRC 10. Therefore, exploring biomarkers for early diagnosis is urgently needed. On the other hand, it is important to seek new therapeutic targets to improve the prognosis of patients with CRC. In recent years, the rapid development of high-throughput sequencing and bioinformatics has provided more opportunities to further understand the mechanism of CRC and explore diagnostic and therapeutic targets 11. Previous studies have shown that TSPEAR is expressed in the liver, intestine, lung, kidney, and testis 7. However, the function of TSPEAR is still unclear, especially in the field of cancer. Thus, the significant role of TSPEAR in the development and progression of tumors needs to be clarified.

In this study, we used bioinformatics and public databases to comprehensively analyze the expression of TSPEAR across cancers and found that TSPEAR was highly expressed in some cancers, especially in CRC. Interestingly, previous studies have shown that TSPE-AS1 plays a protective role in hepatocellular cancer, which indirectly shows that TSPEAR plays a role in promoting hepatocellular cancer. This is consistent with the results of pan-cancer analysis in our study, and TSPEA is highly expressed in hepatocellular carcinoma. These results further indicate that TSPEAR has an important role in promoting cancer. To explore the relationship between TSPEAR and CRC in detail, RNA-seq data and clinical characteristics of CRC in the TCGA database were analyzed systematically. Cox regression analysis indicated that TSPEAR was an independent risk factor for CRC patients. TSPEAR, age, and TNM stage were used to construct a nomogram to predict the prognosis of patients with CRC, and ROC curve analysis showed that the nomogram had certain predictive values at 1, 2, and 5 years. Nomograms are an effective tool for predicting the prognosis of tumor patients 12. The nomogram constructed with TSPEAR, age, and TNM stage showed good predictive power in this study.

The tumor microenvironment includes various immune cells that play an important role in tumor progression, metastasis, and treatment resistance. In our study, we found infiltration of CD8 T cells, T follicular helper cells, activated NK cells, and neutrophils were lower in the high expression group. Previous studies have shown that high levels of CD8^+^ T cells can inhibit the proliferation and invasion of tumor cells 13. T follicular helper cells are a new subset of CD4 helper T cells that can persistently secrete chemokine receptors to exert immune effects 14. In research on triple-negative breast cancer, under the action of immune checkpoint inhibitors, T follicular helper cells can activate B cells to transform into plasmacyte cells, which produce antibodies. If the antibody produced by plasmacyte cells is inhibited, the effect of immunotherapy will be severely limited. The increase in regulatory T cells and natural killer cells in primary CRC suggests a good prognosis for the tumor 15. N1-type neutrophils inhibit tumor growth by directly producing cytotoxic substances or by the antitumor immune response 16. In our study, the high-expression group showed a lower level of immune infiltration, which indicated that a high level of TSPEAR may be related to tumor immunosuppression.

Previous studies have indicated that TMB plays important roles in tumor-infiltrating immune cells and the clinical efficacy of immunotherapy 17. According to our analysis, patients with high TSPEAR expression had a lower TMB than those with low TSPEAR expression. Survival analysis indicated that patients with higher TMB had shorter OS than those with lower TMB. In multiple tumors, a high TMB suggests a poor prognosis for the patient 18. A previous study indicated that the objective remission rate of patients with high TMB increased significantly among patients with CRC treated with PD-1/PD-L1 immune checkpoint inhibitors 19. A high TMB can lead to increased antigen presentation on the surface of tumor cells and increase the immunogenicity of tumors, thereby improving the curative effect of immunotherapy 20. In renal clear cell carcinoma and prostate carcinoma, patients with higher TMB tend to have a poor prognosis 21. In bladder cancer, a higher TMB indicates a longer survival time 22. Wang et al. noted that a high tumor mutation burden indicates a better prognosis in CRC patients with KRAS mutations 23. The influence of TMB on the prognosis of patients differs among different types of tumors. Further study is needed to determine the effect of TMB on the prognosis of CRC.

In this study, KEGG analysis showed that DEGs were enriched in the Wnt signaling pathway and the Hippo signaling pathway. The Wnt signaling pathway plays a vital role in the initiation, progression, and metastasis of CRC 24. The Wnt signaling pathway and PI3K/Akt signaling pathway have synergistic effects on the development and progression of CRC 25. We further investigated the transcription factor associated with TSPEAR. TGIF2 was identified as the transcription factor of TSPEAR, and its binding sequence may be present approximately 241 bp upstream of the TSPEAR gene. We induced TSPEAR silencing by transfecting siRNA into two types of cell lines to investigate the role of TSPEAR in the biological function of CRC cells. The results confirmed that after knocking down TSPEAR, cell proliferation, migration, and invasion were impaired in HT29 and HCT116 cells. Upon knockdown of TGIF2, the mRNA and protein expression of TSPEAR was decreased, which indicated that TGIF2 could regulate the expression of TSPEAR. These results indicated that TSPEAR and TGIF2 are crucial for maintaining the tumor activity of CRC cells in vitro; thus, they may be new targets for the treatment of CRC. TGIF2 is a transcription regulator that is phosphorylated by EGFR/ERK signaling. TGIF2 promotes epithelial-mesenchymal transition and metastasis of lung adenocarcinoma, making it a potential treatment target for lung adenocarcinoma 26. In glioblastoma, TGIF2 promotes tumor progression and is a potential therapeutic target 27.

In hepatocellular carcinoma and tongue squamous cell carcinoma, TSPEAR-AS1 plays a protective role, which indirectly indicates that TSPEAR plays a role in promoting cancer in some cancers 7, 8, 28. Similarly, we found that the high expression of TSPEAR in CRC tissue was related to poor prognosis, and one of its mechanisms might be that the high expression of TSPEAR was related to low immune infiltration. At the same time, this study initially revealed the role of TSPEAR and its transcription factor TGIF2 through in vitro experiments, providing a strong basis for TSPEAR as a therapeutic target. Although our research is the first work to reveal the role of TSPEAR in CRC, it also has some limitations. First, our data comes from the TCGA public database, so further clinical data and clinical samples are needed for verification. Additionally, the role of TSPEAR and TGIF2 was primarily explored through in vitro experiments. Further systematic experiments need to explore the downstream potential mechanism. In vivo experiments are also needed to verify the role of TSPEAR and TGIF2.

## Conclusion

In conclusion, TSPEAR is overexpressed in CRC and is significantly related to a poor prognosis. In addition, TSPEAR expression is significantly associated with tumor immune cell infiltration and TMB, which affect the biological behavior of tumor cells. TGIF2 regulates TSPEAR expression and may be a therapeutic target. This study provided multilevel evidence to indicate the potential function of TSPEAR as a diagnostic biomarker and therapeutic target of CRC.

## Supplementary Material

Supplementary figures and tables.

## Figures and Tables

**Table 1:** Patients' characteristics

Variables	n=590 (%)
Age	
Median (range)	67 (31-90)
Gender	
Male	309 (52.4)
Female	281 (47.6)
Tumor location	
colon	435 (73.7)
rectal	155 (26.3)
pT stage	
T1	19 (3.2)
T2	106 (18.0)
T3	414 (70.2)
T4	51 (8.6)
pN stage	
N0	355 (60.1)
N1	136 (23.1)
N2	99 (16.8)
pM stage	
M0	472 (80.0)
M1	65 (11.0)
Mx	53 (9.0)
Vital status	
Alive	522 (88.5)
Dead	68 (11.5)

## Abbreviations

CRC: Colorectal cancer
TCGA: The cancer genome atlas
GTEx: Genotype-Tissue expression
ROC: Receiver operating characteristic
ssGSEA: Single-sample gene set enrichment analysis
TMB: Tumor mutation burden
AUC: Areas under the ROC curve
OS: Overall survival
DEGs: Differentially expressed genes
DSS: Disease-specific survival
FDR: False discovery rate
FC: Fold change
KEGG: Kyoto Encyclopedia of Genes and Genomes
GO: Gene ontology
KM: Kaplan-Meier
