# Supplementary material for: Comprehensive analysis reveals TSPEAR as a prognostic biomarker in colorectal cancer
Source: J Cancer. 2024 Jan 1;15(3):809–24. doi: 10.7150/jca.90028 (PMC10777046; doi:10.7150/jca.90028)
Supplement: Supplementary file 1 — Supplementary figures and tables. [file jcav15p0809s1.pdf]

Supplementary Material

Supplementary figures and tables.

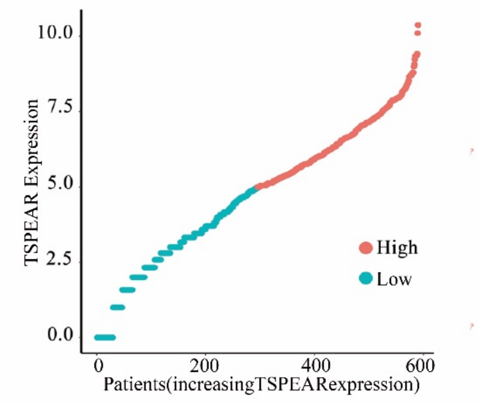

Figure S1. Scatter diagram of TSPEAR mRNA expression from low to high.

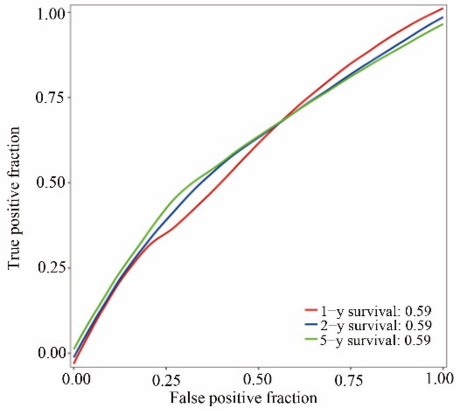

Figure S2. ROC curves of 1-, 2-, and 5- year OS predicted by TSPEAR.

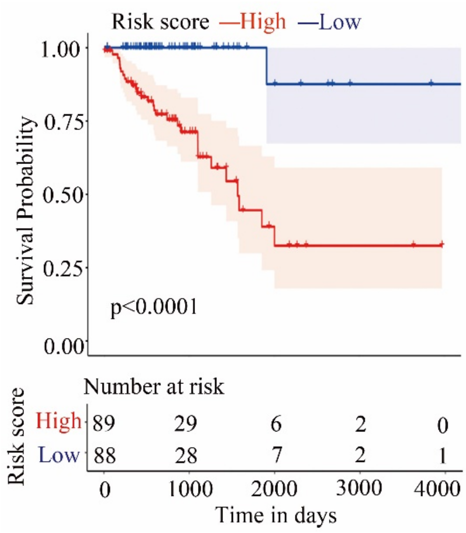

Figure S3. Kaplan–Meier curves of patients with CRC based on the monogram.

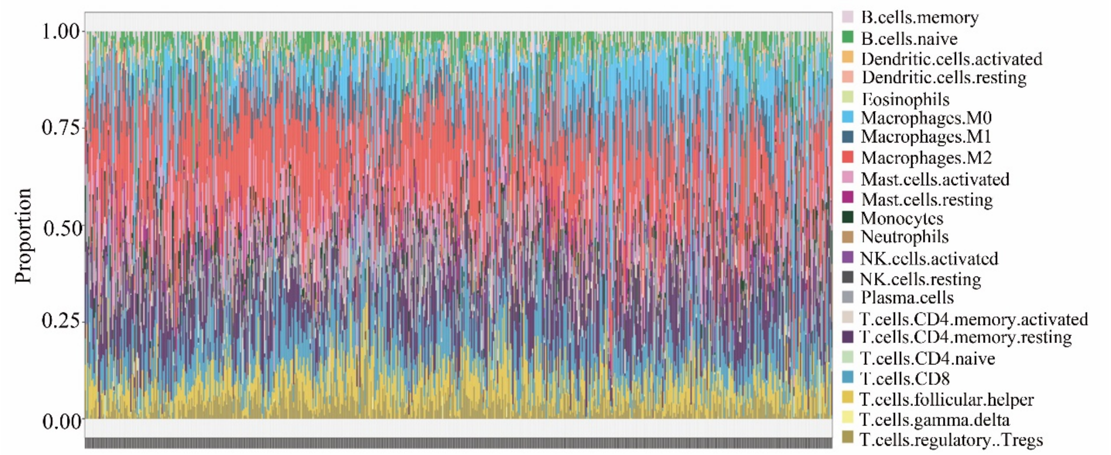

Figure S4. The percentage of immune cells in each sample.

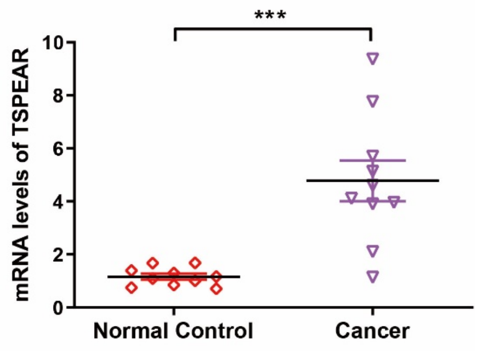

Figure S5. The mRNA expression levels of TSPEAR in CRC tissues and their paired normal tissues.

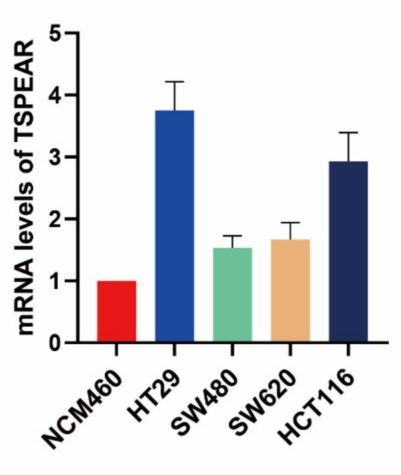

Figure S6. The mRNA expression levels of TSPEAR in normal colonic mucosa cells and CRC cells.

Table S1 Correlation of TSPEAR and transcription factors in TCGA-COADREAD

Transcription factors	Rho	Spearman P-value
TCF7	0.446573829	5.65E-28
PROX1	0.441785275	2.39E-27

MLXIPL	0.431424441	5.04E-26
PLAGL2	0.423542234	4.77E-25
GTF2IRD1	0.414284685	6.22E-24
TGIF2	0.401765249	1.76E-22
ZNF74	0.397026456	6.04E-22
ASCL2	0.387151019	7.35E-21
ZBTB12	0.384433931	1.44E-20
ZC3H8	0.379693087	4.60E-20
ZNF316	0.376476364	9.99E-20
MYT1	0.373364062	2.10E-19
HIF3A	0.36970287	4.98E-19
KCNIP3	0.369261725	5.52E-19
SP6	0.365980381	1.18E-18
TCF3	0.360630086	4.03E-18
ZNF251	0.360590951	4.07E-18
ASCL5	0.360237135	4.41E-18
ZNF703	0.356041404	1.13E-17
ARID3A	0.353317805	2.08E-17
CXXC5	0.353276202	2.09E-17
VENTX	0.353224945	2.12E-17
ZFP69B	0.353094201	2.18E-17
ZNF692	0.348581287	5.86E-17
DLX3	0.34670611	8.80E-17
MYC	0.346297627	9.62E-17
L3MBTL1	0.344478399	1.42E-16
DLX4	0.343643152	1.70E-16
FOXO6	0.343571253	1.73E-16

NKRF	0.343218688	1.86E-16
TFAP4	0.340640675	3.22E-16
DACH1	0.338199572	5.38E-16
TIGD1	0.335038671	1.04E-15
ZNF280C	0.333736369	1.36E-15
PBX1	0.33168451	2.07E-15
TCF7L1	0.329538253	3.21E-15
SOX4	0.326258738	6.22E-15
ZNF473	0.323310289	1.12E-14
VDR	0.322286878	1.37E-14
ZNF485	0.319596605	2.33E-14
POU5F1B	0.319140681	2.54E-14
FOXN3	0.312268356	9.56E-14
SOX9	0.312252789	9.59E-14
KLF8	0.310179042	1.42E-13
ZBTB7B	0.306822258	2.67E-13
ZFP3	0.306597171	2.78E-13
TEAD4	0.30558684	3.35E-13
HES6	0.305006103	3.74E-13
TBX10	0.303244397	5.17E-13
PBX4	0.302947393	5.46E-13
FOXP4	0.300428962	8.65E-13

Table S2 Prediction of transcription factor binding motifs in TSPEAR by FIMO

motif_alt_id	sequence_name	start	stop	p-value	q-value
PLAGL2	hg38_knownGene_ENST00000323084.9	2950	2959	4.02E-06	0.0178
PLAGL2	hg38_knownGene_ENST00000323084.9	2951	2960	1.53E-05	0.0226

PLAGL2	hg38_knownGene_ENST00000323084.9	2951	2960	1.53E-05	0.0226
PLAGL2	hg38_knownGene_ENST00000323084.9	2952	2961	9.32E-05	0.103
TGIF2	hg38_knownGene_ENST00000323084.9	2759	2770	3.00E-05	0.177
